# Genetic Complexity of Sinoatrial Node Dysfunction

**DOI:** 10.3389/fgene.2021.654925

**Published:** 2021-04-01

**Authors:** Michael J. Wallace, Mona El Refaey, Pietro Mesirca, Thomas J. Hund, Matteo E. Mangoni, Peter J. Mohler

**Affiliations:** ^1^Frick Center for Heart Failure and Arrhythmia Research, The Ohio State University Wexner Medical Center, Columbus, OH, United States; ^2^Dorothy M. Davis Heart and Lung Research Institute, The Ohio State University Wexner Medical Center, Columbus, OH, United States; ^3^Department of Physiology and Cell Biology, College of Medicine, The Ohio State University Wexner Medical Center, Columbus, OH, United States; ^4^CNRS, INSERM, Institut de Génomique Fonctionnelle, Université de Montpellier, Montpellier, France; ^5^Laboratory of Excellence ICST, Montpellier, France; ^6^Department of Biomedical Engineering, College of Engineering, The Ohio State University, Columbus, OH, United States; ^7^Division of Cardiovascular Medicine, Department of Internal Medicine, College of Medicine, The Ohio State University Wexner Medical Center, Columbus, OH, United States

**Keywords:** genetics, sick sinus syndrome, sinoatrial node dysfunction, atrial fibrillation, calsequestrin-2, GIRK4, HCN4, Na_v_1.5

## Abstract

The pacemaker cells of the cardiac sinoatrial node (SAN) are essential for normal cardiac automaticity. Dysfunction in cardiac pacemaking results in human sinoatrial node dysfunction (SND). SND more generally occurs in the elderly population and is associated with impaired pacemaker function causing abnormal heart rhythm. Individuals with SND have a variety of symptoms including sinus bradycardia, sinus arrest, SAN block, bradycardia/tachycardia syndrome, and syncope. Importantly, individuals with SND report chronotropic incompetence in response to stress and/or exercise. SND may be genetic or secondary to systemic or cardiovascular conditions. Current management of patients with SND is limited to the relief of arrhythmia symptoms and pacemaker implantation if indicated. Lack of effective therapeutic measures that target the underlying causes of SND renders management of these patients challenging due to its progressive nature and has highlighted a critical need to improve our understanding of its underlying mechanistic basis of SND. This review focuses on current information on the genetics underlying SND, followed by future implications of this knowledge in the management of individuals with SND.

## Introduction

Automaticity is the primary function of the sinoatrial node (SAN). SAN pacemaker cells have the shortest depolarization phase of the action potential and the fastest firing rate, making the SAN the dominant pacemaker of the heart (Kennedy et al., 2016). The SAN initiates an electrical impulse that propagates throughout the heart, establishing a normal heart rhythm. The sinoatrial node is surrounded by a collagen frame, which helps protect SAN cells against ectopic activation from other atrial cells (James, 1977; Kalyanasundaram et al., 2019). The SAN location was initially reported near the epicardium between the superior vena cava and the right atrium in the posterior right atrial wall (Keith and Flack, 1907). The human SAN was later described to commonly exist laterally and inferior to the terminal crest. Infrequently, the SAN can exist at the junction between the superior caval vein and right atrium, or even across the terminal crest into the interatrial groove (Anderson et al., 1979; Sanchez-Quintana et al., 2005; Dobrzynski et al., 2007). Recently, evidence has suggested the simultaneous existence of superior and inferior SAN in the human heart located near the superior and inferior venae cavae, respectively. The superior SAN is suggested to generally control higher heart rate, while the inferior (“back-up”) SAN is suggested to control lower heart rate. Optical mapping data shows that the heart rate control distinctly jumps between cluster control, rather than a gradient transition, during physiologic heart rate changes (Brennan et al., 2020). The SAN primarily contains three specialized cell types: pale cells (P cells), transitional cells (T cells), and recently described “fibroblast-like” cells, intertwined in collagen, fibroblasts, fatty tissue, nerves, and capillaries (James, 1977; Balbi et al., 2011; Csepe et al., 2015). P cells are localized to the central region of the SAN and have a single membrane with very little membrane specialization resulting in poorly defined intercalated discs, few desmosomes, and few gap junctions (Ho and Sánchez-Quintana, 2016). Gap junctions are rare in P cells, and junctions are only formed between P cells or T cells. P cells do not form junctions with other atrial myocytes (James, 1977). Automaticity arises from P cells (James et al., 1966; Woods et al., 1976). Interestingly, transitional cells, located in the periphery of the SAN, act as the distributors of action potentials to atrial myocardium and internodal conduction pathways (Boyett et al., 2000). T cells are a loosely defined, varied group of cells. Many T cells look like P cells with reduced myofibrils, while others closely resemble atrial myocytes (James et al., 1966). Finally, the function of “fibroblast-like” cells has yet to be defined, but presumably, these cells play a role in maintaining the structure of the SAN (Balbi et al., 2011). The SAN was initially proposed to propagate action potentials to the atrioventricular (AV) node via three tracts: the anterior (which splits into two bundles), middle, and posterior internodal tracts (James, 1963; Kennedy et al., 2016). However, recent studies failed to confirm this hypothesis. Interestingly, current optical mapping studies using rat and human atrial tissues suggest the presence of more than a single definite pacemaker site. Overall, the existence of clearly defined intermodal tracts has not been confirmed via optical and electrical mapping (Boineau et al., 1988; Brennan et al., 2020).

The SAN receives blood flow through the sinus node artery, the largest atrial coronary branch, which originates from the left or right coronary artery (Verhaeghe and van der Hauwaert, 1967). Of note, parasympathetic modulation of the heart is primarily through the vagal postganglionic pathways along the *sulcus terminalis* in the subepicardial region adjacent to the SAN artery (Bluemel et al., 1990). Innervation is both parasympathetic and sympathetic to the SAN, yet neither innervation pathway has any direct contact with P or T cells (Balbi et al., 2011). The intrinsic cardiac nervous system is mainly composed of cardiac ganglia that play a significant role in the integration of cardiac electrophysiology (Fedele and Brand, 2020).

Interestingly, it has been suggested that the total amount of SAN cells is inversely proportional to the age of an individual, suggesting that the number of SAN cells and the volume of SAN cells decrease over time (Thery et al., 1977; Shiraishi et al., 1992). However, Alings et al. (1995) found that the SAN does not change in dimensions during an entire adult life span. Although relative collagen increases from childhood to adulthood, collagen levels do not change once adulthood is reached. Instead, the structure of the collagen changes throughout adulthood (Alings et al., 1995).

SND occurs in one of every 600 cardiac patients above the age of 65 (Rodriguez and Schocken, 1990). Symptomatic SND is the most common rationale for permanent pacemaker implantation (Kusumoto et al., 2019). In general, “sick sinus syndrome” (SSS), sinus node dysfunction (SND), and sinoatrial node dysfunction (also SND) are used interchangeably in the literature. SND is usually accompanied by structural abnormalities. Symptoms of SND include sinus bradycardia [heart rate less than 60 beats per minute (bpm)], sinus arrest (total absence of atrial or ventricular activity), SA block, and bradycardia/tachycardia syndrome (cycling between supraventricular tachyarrhythmias and sinus bradycardia) (Alpert and Flaker, 1983). Syncope has also been reported by about half of patients with SND (Menozzi et al., 1998; Brignole et al., 2013). Although structural defects accompany most forms of SND, syncope and bradycardia associated with SND have also been observed in patients with normal cardiac anatomy (Yabek et al., 1982). SND is generally associated with impaired pacemaker function causing abnormal heart rhythm, but SND can also be associated with impulse transmission issues from apoptosis in the AV node, sinus node, and internodal pathways, which may cause death from complete heart block (James et al., 1996). Further, blockage of the sinus node artery preventing blood flow to the SAN cells can result in SND and sudden death (Jing and Hu, 1997).

This review focuses on current information on the genetics underlying SND, followed by a brief overview of the recent update in clinical management of patients with SND. A better understanding of the genetics and the molecular mechanisms of the SAN and SND will improve current diagnostic measures and identify alternative therapeutic approaches. The genes currently implicated in human SND are summarized in Table 1. Although not the focus of this review, SND is also commonly a secondary symptom to other systemic and cardiovascular conditions. Congestive heart failure in particular results in SAN remodeling and a reduction in resting heart rate, which can result in SND (Sanders et al., 2004). Atrial fibrillation (AF) and atrial tachycardia, cardiac transplantation, drug toxicity, hyperinsulinemia and insulin resistance (diabetes type II), sinus node artery obstruction, hyperparathyroidism, intracranial conditions, epilepsy, myxedema coma, cardiac lymphoma, infections, and myocardial infarction are all potential procedures, diseases, or conditions that can result in secondary SND (Lown, 1967; Bexton et al., 1984; Bigger and Sahar, 1987; Wasada et al., 1995; Hasdemir et al., 2003; Shah et al., 2004; Dadlani et al., 2010; Ravindran et al., 2016; Kondo et al., 2020; Kousa et al., 2020; Mesirca et al., 2020). Further, exercise can also highlight previously unreported SND. Chronotropic intolerance, or the inability of heart rate to properly respond to stress and exercise, is a common first indicator of symptomatic SND (de Ponti et al., 2018).

**TABLE 1:** Proteins implicated in human SND.

**Protein**	**Gene**	**Associated cardiac diseases**	**References**
Calsequestrin-2	*CASQ2*	SND/bradycardia, catecholaminergic polymorphic ventricular tachycardia (CPVT), atrial arrhythmias	Reid et al., 1975; Leenhardt et al., 1995; Sumitomo et al., 2007; Weeke et al., 2014; Glukhov et al., 2015
Ryanodine receptor 2	*RYR2*	SND/bradycardia, CPVT, atrial arrhythmias	Sumitomo et al., 2007; Domingo et al., 2015; Miyata et al., 2018; Dharmawan et al., 2019
G protein-activated inward rectifier potassium channel 4	*KCNJ5*	SND/bradycardia, atrial arrhythmias, long QT syndrome type 13, Andersen–Tawil syndrome	Dobrev et al., 2001; Zhang et al., 2009; Yang et al., 2010; Wang et al., 2013; Kokunai et al., 2014; Kuß et al., 2019
Guanine nucleotide-binding protein subunit beta-2/5	*GNB2/GNB5*	SND/bradycardia, cognitive disability, cardiac conduction abnormalities	Lodder et al., 2016; Stallmeyer et al., 2017; Fukuda et al., 2020
Sodium/calcium exchanger 1	*SLC8A1*	Conduction disorders (PR and QT prolongation), ventricular arrhythmias, Kawasaki disease	Kim et al., 2012; Hong et al., 2014
Sodium voltage-gated channel alpha subunit 5	*SCN5A*	SND/bradycardia, long QT syndrome type 3, Brugada syndrome, dilated cardiomyopathy, conduction disorders, infant death syndrome	van den Berg et al., 2001; Benson et al., 2003; McNair et al., 2004; Smits et al., 2005; Nakajima et al., 2013; Freyermuth et al., 2016; Yang et al., 2017; Denti et al., 2018
Hyperpolarization activated cyclic nucleotide gated potassium channel 4	*HCN4*	SND/bradycardia, ventricular arrhythmias, left ventricular non-compaction	Schulze-Bahr et al., 2003; Ueda et al., 2004; Nof et al., 2007; Milano et al., 2014
Ankyrin-B	*ANK2*	SND/bradycardia, CPVT, atrial arrhythmias, arrhythmogenic cardiomyopathy	Curran and Mohler, 2011; Roberts et al., 2019
Myosin heavy chain 6	*MYH6*	SND/bradycardia, aorta coarctation, ventricular arrhythmias	Holm et al., 2011; Lam et al., 2015; Bjornsson et al., 2018
Lamin A	*LMNA*	SND/bradycardia, dilated cardiomyopathy, conduction disorders	MacLeod et al., 2003; Zaragoza et al., 2016; Lin et al., 2018; Yokokawa et al., 2019
L-type calcium channel subunit Ca_v_1.3	*CACNA1D*	Sinoatrial node dysfunction and deafness (SANDD)	Baig et al., 2011; Liaqat et al., 2019
Short-stature homeobox 2	*SHOX2*	SND/bradycardia, atrial arrhythmias	Hoffmann et al., 2016, 2019; Li et al., 2016

One would be remiss to not specifically mention the recently uncovered link between the novel coronavirus 2019 (COVID-19) disease and SND. COVID-19, caused by severe acute respiratory syndrome coronavirus-2 (SARS-CoV-2), may cause various cardiovascular problems even without preexisting cardiac conditions (Clerkin et al., 2020; World Health Organization, 2020). In May 2020, COVID-19 was first reported to cause SND in two patients. Both patients were older than 70 years, and both experienced sinus bradycardia following intubation for acute hypoxic respiratory failure. Each patient still experienced SND 2 weeks after onset (Peigh et al., 2020). Two COVID-19-positive patients were further reported to suffer from AV dysfunction and SND without a history of arrhythmias (Babapoor-Farrokhran et al., 2020). The reported cases suggest an interesting association between COVID-19 and SND as a secondary symptom.

## Genetics of Sinoatrial Node Dysfunction

**FIGURE 1:**
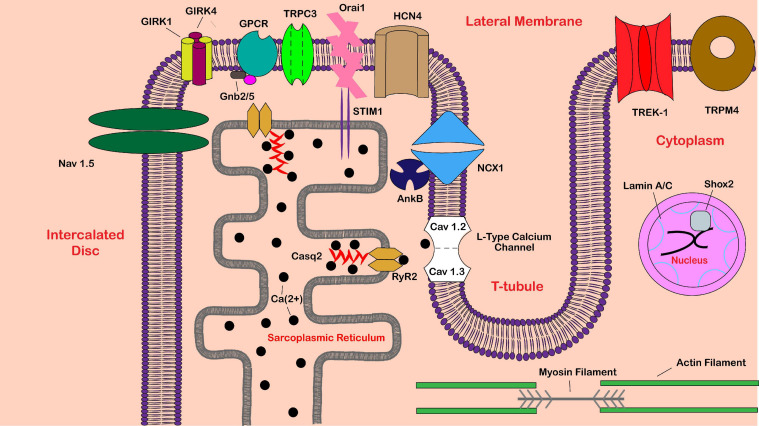
Schematic of proteins implicated in sinoatrial node dysfunction. Shown is a partial sinoatrial (SA) pacemaker cell. Proteins are labeled in black text, while various cellular locations are labeled in red text. Abbreviations include calsequestrin-2 (Casq2), ryanodine receptor 2 (RyR2), G protein-activated inward rectifier potassium channel 1/4 (GIRK1/4), guanine nucleotide-binding protein subunit beta-2/5 (Gnb2/5), G protein-coupled receptor (GPCR), sodium/calcium exchanger 1 (NCX1), voltage-gated sodium channel alpha subunit 5 (Na_v_ 1.5), hyperpolarization activated cyclic nucleotide gated potassium channel 4 (HCN4), ankyrin-B (AnkB), short-stature homeobox 2 (Shox2), transient receptor potential cation channel subfamily C member 3 (TRPC3), stromal interaction molecule 1 (STIM1), calcium-release-activated calcium channel protein 1 (Orai1), potassium two pore domain channel subfamily K member 2 (TREK-1), and transient receptor potential melastatin 4 (TRPM4).

SND mainly affects the older population, although it can affect people at any age. Most cases of SND are not inherited. However, several genes coding for ion channels such as *HCN4* and *SCN5A*, cytoskeletal proteins, and proteins intricate to cardiac development have been linked to SND (de Ponti et al., 2018). Gene products associated with SND in the SAN are shown in Figure 1. Major genes linked to SND etiology, as well as human studies and animal models used to characterize the SND phenotype, will be discussed below. Mouse models are a common tool for *in vivo* studies of various cardiac proteins and genes. Although heart size and heart rate are significantly different between humans and mice, the human and murine hearts develop and function similarly (Wessels and Sedmera, 2003). Still, comparisons between the two species are not perfect. Note that many mouse models are full body or cardiac-specific knockouts of a gene, while human gene variants most commonly only have a single nucleotide change, which may still result in a fully transcribed protein. CRISPR/Cas9 technology, which allows the development of more humanized animal models via induction of specific point mutations, remains one of the greatest tools for improving translational animal studies going forward.

Genetic complexity and pleiotropy remain a big challenge to proper SND/SSS diagnosis with likely multiple complexities involved including genetic variation, underlying conditions, and environmental factors. With numerous SA-expressed gene variants associated with SND, and only moderate penetrance, SND can continue within families for generations before diagnosis (Abe et al., 2014). Further, compound variants for prominent SND-implicated genes seem to increase penetrance, emphasizing SND as an oligogenic disease (Baskar et al., 2014; de Filippo et al., 2015; Sacilotto et al., 2017).

### Calsequestrin-2 (*CASQ2*) and Ryanodine Receptor 2 (*RYR2*)

#### Background

Cardiac calsequestrin, calsequestrin-2 (Casq2), localized to the sarcoplasmic reticulum (SR), is a low-affinity, high-capacity, Ca^2+^-binding protein involved in the ability of the SR to preserve and release Ca^2+^ in cardiac myocytes (Murphy et al., 2011). Voltage-gated Ca^2+^ channels (L-type calcium channels) create an initial Ca^2+^ influx into the cell, which causes RyR2 receptors to release more calcium from the SR in a process called calcium-induced calcium release. Calsequestrin, the major Ca^2+^-binding protein in the SR, is anchored to the membrane of the SR by RyR2 (either directly or indirectly via triadin and junctin) and regulates the release of Ca^2+^ through the RyR2 channel (Beard et al., 2004). Importantly, calsequestrin, the ryanodine receptor, junctin, and triadin are all necessary for proper calcium cycling (Zhang et al., 1997).

#### Human Studies

Loss-of-function variants in *CASQ2* can result in catecholaminergic polymorphic ventricular tachycardia (CPVT), bradycardia, and atrial arrhythmias (Glukhov et al., 2015). CPVT was first identified in 1975 as a case report of “bidirectional tachycardia” induced by physical effort and stress in a 6-year-old female with no structural abnormalities (Reid et al., 1975). A later follow-up of 21 children over 7 years properly emphasized CPVT as a separate entity of ventricular tachycardia in children, emphasizing the dangers of misdiagnosis with the lurking potential for spontaneous sudden cardiac death (SCD) without immediate therapy. Leenhardt et al. (1995) showed the adrenergic-induced ventricular arrhythmias to be preventable with proper β-blocker treatment.

The CPVT phenotype from *CASQ2* variants is attributed to an increase in expression of calreticulin and RyR2 (Song et al., 2007). CPVT is also commonly induced by *RYR2* variants and has been highly associated with bradycardia (Miyata et al., 2018). An exon 3 deletion of *RYR2* (c.169-353_273 + 657del) was found in a family with a history of SND and CPVT (Dharmawan et al., 2019). Exon 3 deletions were identified in two additional families that showed symptoms of SND (Bhuiyan et al., 2007). Further, a RyR2 (R420Q) missense mutation was associated with sinus bradycardia and atrial arrhythmias (Domingo et al., 2015). Although CPVT is well documented across *CASQ2* and *RYR2* variants, SAN dysfunction and atrial arrhythmias have been identified in CPVT patients, signifying the importance of calsequestrin-2 and ryanodine receptor 2 in proper SAN functioning (Sumitomo et al., 2007). Additionally, a study across 303 patients with lone AF found rare *CASQ2* variants, implicating *CASQ2* in AF as well (Weeke et al., 2014).

#### Mouse Models

Glukhov et al. (2015) described the first *Casq2*-null (*Casq2*^–/–^) mouse model with SAN defects. The phenotype included bradycardia, RR interval variability, SAN conduction abnormalities, and abnormally high atrial ectopic activity resulting in AF, implicating calsequestrin-2 in SAN functioning. Increased fibrosis in the pacemaking complex was observed in *Casq2*-deficient mice. At the cellular level, lack of functioning *Casq2* caused abnormal calcium release from the SR and increased diastolic calcium concentration, resulting in a delay between action potential and transient calcium upstrokes (Glukhov et al., 2015). This disruption in intracellular calcium cycling explains the SAN, atrial myocyte, and ventricular myocyte dysfunction seen in individuals with *CASQ2* loss-of-function variants.

Atrial burst pacing induced atrial flutter and AF in *Casq2*^–/–^ mice versus wild-type (WT) mice, and isolated *Casq2*-deficient hearts showed ectopic foci from the pulmonary vein region when visualized with atrial optical voltage maps. Isolated hearts from *Casq2*-null mice also experienced diastolic subthreshold spontaneous Ca elevations (SCaEs) and delayed afterdepolarizations (DADs) when AF was not reached, but *R*-propafenone (RyR2 and Na^+^ channel blocker) was shown to prevent AF, DADs, and SCaEs in these mice. The authors attributed this success mostly to the inhibition of RyR2, as an equipotent Na_v_ channel inhibitor did not reach the same result (Faggioni et al., 2014). Notably, atrial overdrive pacing suppressed ventricular arrhythmias in a *Casq2*-null mouse model and therefore could provide a new therapeutic option for CPVT (Faggioni et al., 2013). Interestingly, a RyR2 R4496C homozygous mouse showed sinus pausing, atrial arrhythmias, and reduced SAN automaticity. This variant is associated with enhanced RyR2 activity and supports a link between *RYR2* variants in humans with SND (Zhang et al., 2013). In general, loss-of-function *Casq2* variants and gain-of-function *RyR2* variants both contribute to unreliable and irregular calcium cycling in the SAN, atria, and ventricles. This likely translates to human variants and provides some insight into the cause of CPVT, AF, and SND seen in carriers of *CASQ2* and *RYR2* variants.

### G Protein-Activated Inward Rectifier Potassium Channel 4 (*KCNJ5*)

#### Background

Activation of G protein via a transmitter is a common type of cell–cell communication. In general, a neurotransmitter binds to a seven transmembrane receptor on the outside of the cell, which results in the exchange of GDP for GTP on the inward side of the receptor, allowing the dissociation of the heterotrimeric G-protein subunits, which then further act as effectors (Gilman, 1987; Neer, 1995; Mark and Herlitze, 2000). For heart rate modulation, the Gβγ (as opposed to the Gα) subunit specifically activates the *K*_*Ach*_ channel by binding to the N- and C-termini of GIRK1 (G protein-regulated inwardly rectifying *K*^+^) and GIRK4 subunits directly (Logothetis et al., 1987; Huang et al., 1995). The muscarinic acetylcholine *K*^+^ channel (*K*_*Ach*_) is composed of GIRK1 and GIRK4 (Kir3.4, *KCNJ5* gene) subunits in the atria and contributes to heart rate regulation in mammals (Wickman et al., 1998; Mark and Herlitze, 2000). During parasympathetic stimulation, *K*_*Ach*_ channels are activated, slowing the heart rate and the contractile force of the heart (Wickman et al., 1997). Girk4 knockout mice lacked not only *I*_*KAch*_ but also Girk1 expression, reinforcing that Girk4 plays a leading role in the expression and localization of Girk1 to the cell membrane (Wickman et al., 1998; Kennedy et al., 1999). The *K*_*Ach*_ channel is rapidly and reversibly inhibited upon membrane stretch that allows mechano-electrical regulation of the atria (Ji et al., 1998). Although Girk4 is necessary for proper Girk1 membrane expression, Girk4 homotetramers have also been identified in the atria (Corey and Clapham, 1998; Bender et al., 2001). Regulation of the Girk1/Girk4 heterotetrameric channel occurs via phosphorylation by cyclic AMP-dependent protein kinase A (PKA) (upregulation) and via dephosphorylation by protein phosphatase 2A (PP2A) (downregulation) (Müllner et al., 2003).

Remarkably, the guanine nucleotide-binding protein subunits 2 and 5 (GNB2 and GNB5), which create the beta subunits of the G protein that interacts with GIRK1 and GIRK4, also play a role in SND. *GNB5* variants have been reported in a patient with sinus bradycardia and cognitive disability (Lodder et al., 2016). A *GNB2* variant was more strictly associated with cardiac conduction abnormalities resulting in SND and AV block (Stallmeyer et al., 2017).

#### Human Studies

Dobrev et al. (2001) compared 24 patients with chronic AF and aberrant atrial pacemaking function with 39 patients with normal sinus rhythm. The density of *I_*K*__*Ach*_* in patients with chronic AF was only 50% of the density of *I*_*KAch*_ in the sinus rhythm group. GIRK4 mRNA expression was also decreased, potentially implicating reduced GIRK4 expression in increased atrial excitability (Dobrev et al., 2001). C171T and G810T variants in GIRK4 have now been identified as risk factors for lone paroxysmal AF in Chinese populations (Zhang et al., 2009). Further, A G387R heterozygous dominant variant in GIRK4 was found in a large Chinese kindred with clinical long QT syndrome (LQTS), a hereditary disorder that leads to SCD. In 2019, a GIRK4 variant (W1010C) was first identified in a three-generation family with SND. The W1010C variant in GIRK4 interestingly resulted in increased *I*_*KAch*_. This gain-of-function variant caused an enhanced parasympathetic tone, causing familial SND and hyperpolarization of the pacemaker cells (Kuß et al., 2019). Overall, loss-of-function variants seem to induce atrial arrhythmias, while gain-of-function variants may be implicated in SND.

#### Mouse Models

SAN pacemaker cells from *Kcnj5*^–/–^ mice completely lacked *I*_*KAch*_ and showed about 50% reduction in cholinergic regulation of heart rate (Wickman et al., 1998; Mesirca et al., 2013), further supporting the importance of the GIRK4 in parasympathetic modulation. In addition, *Kcnj5* knockout mice had a 10% increase in resting heart rate with this decreased parasympathetic tone. The ability of these mice to recover to resting heart rate after stress (exercise or pharmacological stimulation) was significantly prolonged (Mesirca et al., 2013). Overall, complete lack of *Kcnj5* expression attenuates the parasympathetic tone and results in an increased HR. Population-wide variants should be replicated in mice to further study the specifics of the mutations, and their effect on the functionality of the GIRK4 protein overall.

### Voltage-Gated Sodium Channel Alpha Subunit 5 (*SCN5A*)

#### Background

Atrial, ventricular, and Purkinje myocyte depolarization, which causes a complete contraction of the heart, is initially regulated by the cardiac sodium channel. Na_v_1.5 (the pore-forming, ion-conducting α-subunit of the cardiac sodium channel) is encoded by *SCN5A*, and variants in this gene have been implicated in a wide range of cardiac diseases such as Brugada syndrome, LQTS, AF, SND, dilated cardiomyopathy, and others (Wilde and Amin, 2018). Butters et al. (2010) analyzed electrophysiological mathematical models of SAN cells, 2D models of the intact SAN-atrium tissue, and actual recordings of activation patterns from isolated intact rabbit SAN-atrium tissue to elucidate the mechanism of *Scn5a*^+/–^ variants on SAN function. Isolated SAN cells from adult rabbits harboring a heterozygous *Scn5a* variant display slower pacemaking rates in the peripheral cells, but not in the SAN central cells, yet 2D models show intact atrium-SAN tissue to have a decreased pacemaking rate overall with AP conduction issues, which may potentially cause SAN exit block and sinus arrest, as seen in SND (Butters et al., 2010).

#### Human Studies

A *SCN5A* variant associated with LQTS type 3 (LQTS3) and Brugada syndrome was first identified in a large family in 2001. Individual carriers within the family that harbored the variant displayed a lower heart rate and experienced marked QT prolongation during episodes of bradycardia, which resulted in sudden death within the family (van den Berg et al., 2001). Further, *SCN5A* human variants (usually autosomal recessive inheritance patterns) were linked to SND (Benson et al., 2003), dilated cardiomyopathy, conduction disorders (McNair et al., 2004; Freyermuth et al., 2016; Yang et al., 2017), and infant death syndrome (Denti et al., 2018).

A loss-of-function E161K Na_v_1.5 variant was identified in two unrelated individuals with family history of bradycardia, SND, conduction disease, and Brugada syndrome. The reduced functional Na_v_1.5 protein expression caused atrial, ventricular, and SAN conduction slowing. Diastolic depolarization rate and upstroke velocity were both reduced in E161K computational models (Smits et al., 2005). An L1821fs/10 *SCN5A* variant causing a C-terminus truncation was identified in a 12-year-old male diagnosed with SND; and when expressed in HEK-293 cells, Na_v_1.5 current density was decreased by 90% (Tan et al., 2007). The SND phenotype identified in *SCN5A* variants seems to generally come secondary to Brugada syndrome and LQTS3. For example, a loss-of-function E1784K variant was identified in 41 individuals, and 39% of individuals had SND, while nearly all of them (93%) had LQT3 (Makita et al., 2008). However, some *SCN5A* variants have shown an SND phenotype without the Brugada-type ST elevation (Nakajima et al., 2013; Wilders, 2018; Alkorashy et al., 2020). An I230T homozygous Na_v_1.5 variant was found in four children with SND, yet heterozygous carriers of the variant showed normal conduction (Neu et al., 2010). Additionally, a case report found an R121W Na_v_1.5 novel variant in an individual diagnosed with SND (Holst et al., 2010). Finally, D349N and D1790N autosomal recessive variants in Na_v_1.5 were associated with pediatric sinus arrest and SND (Kodama et al., 2013).

#### Mouse Models

Mice heterozygous for functional Na_v_1.5 (*Scn5a*^+/−^) demonstrated bradycardia and SA block due to slowed pacemaker rates and slower SA conduction, particularly in larger peripheral SAN cells (Lei et al., 2005). Interestingly, *Scn5a*^+/−^ mice showed sex-dependent effects on SAN functioning in older mice, particularly shown as PR interval prolongation (in old males), RR interval prolongation (longer in old males), QTc prolongation (similar across both genders), and T-wave prolongation (longer in old males) (Jeevaratnam et al., 2010). *Scn5a*^+/−^ mice also had decreased heart rate variability, reduced SAN automaticity, slowed SA conduction, increased fibrosis, and increased fibroblasts as consequences of the decreased Na_v_1.5 expression, particularly in older-age mice (Royer et al., 2005; Hao et al., 2011).

### Hyperpolarization Activated Cyclic Nucleotide Gated Potassium Channel 4 (*HCN4*)

#### Background

The ability of the SAN cells to spontaneously initiate electrical impulse comes from the funny current (*I*_*f*_) activation, a Na^+^/K^+^ depolarization current. Funny current channels have inward current at diastolic voltages that are then activated by membrane hyperpolarization (“membrane clock” hypothesis for pacemaker automaticity) via binding to the intracellular cAMP that can be modified by sympathetic and parasympathetic transmitters, therefore modulating the heart rate (DiFrancesco, 1993; Accili et al., 2002; DiFrancesco, 2010). *I*_*f*_ may also be regulated by cAMP-activated PKA in the SAN (Liao et al., 2010). While there are four members of the HCN channel family, many studies proposed that only HCN2 and HCN4-based channels are expressed in the murine SAN, with HCN4-based channels having a higher level of expression, confirming the role of HCN4-based channels in driving cardiac pacemaker activity (Moosmang et al., 2001; Xiao et al., 2010). However, Fenske et al. (2013) found HCN1-based channels highly expressed in the SAN and reported HCN1-based channels as critical for the stabilization of the lead pacemaker region in mice. HCN4 channels and beta-2 adrenergic receptors (β2-AR) form a complex that is essential for HCN4 channel regulation (Greene et al., 2012). Interestingly, the expression of HCN2 and HCN4 channels was shown to decrease at the SAN and to increase in the atria and pulmonary vein in older age in dogs, which could account for the disproportionately older population afflicted by SND (Li et al., 2014; Du et al., 2017).

#### Human Studies

In 2003, an *HCN4* variant (573X) causing truncation of HCN4 C-terminus was first identified in a patient with SND, presented as sinus bradycardia and chronotropic incompetence (Schulze-Bahr et al., 2003). Importantly, familial sinus bradycardia linked to C-terminus truncation and loss of cAMP-dependent regulation of HCN4 was documented in 2010 (Schweizer et al., 2010). A D553N HCN4 missense variant was identified in an individual with recurring syncope, QT prolongation, polymorphic VT, and torsade de pointes. The individual variant, when transfected in COS7 cells, displayed a reduction in HCN4 expression at the cellular membrane, correlating the variant and the loss-of-function HCN4 with SND (Ueda et al., 2004). Sixteen family members carrying a G480R missense variant in HCN4 (autosomal dominant) experienced sinus bradycardia. Molecular studies showed reduced synthesis and trafficking of G480R variant HCN4 to the membrane (Nof et al., 2007). A G482R HCN4 variant was reported in a family presenting with bradycardia and left ventricular non-compaction cardiomyopathy (NCCM). Interestingly, this was one of the first studies to link HCN4-associated SND with structural abnormalities of the myocardium (Milano et al., 2014). Schweizer et al. (2014) also identified a family with NCCM and SND carrying a G482R variant in HCN4 along with a cysteine and glycine-rich protein 3 (CSRP3) W4R variant in 2014 (Schweizer et al., 2014). Further, HCN4-R393H loss-of-function, c.1737 + 1 G > T splice site, I1479V loss-of-function, A485E loss-of-function, R375C loss-of-function, and V759I loss-of-function variants have all been identified in individuals with SND and other related cardiac conduction disorders (Hategan et al., 2017; Ishikawa et al., 2017; Servatius et al., 2018; Alonso-Fernández-Gatta et al., 2020; Erlenhardt et al., 2020).

#### Mouse Models

Hcn2-deficient mice presented spontaneous absence seizures and abnormal cardiac sinus rhythm, implicating the HCN family in SAN function (Ludwig et al., 2003). Mice expressing the dominant negative 573X HCN4 isoform lacking cAMP regulation present with SAN bradycardia, similar to that observed in the SND individual (Alig et al., 2009). Conditional knockout of *Hcn4* has produced variable outcome phenotype, from sinus pauses (Herrmann et al., 2007) to severe heart block and death (Baruscotti et al., 2011). A murine tamoxifen-inducible, cardiac-specific knockout model of exon 2 of *Hcn4* channels showed remarkable bradycardia (50% reduction in heart rate), AV block, and death on day 5 on average. Importantly, *I*_*f*_ was reduced by about 70% in these mice (Baruscotti et al., 2011). A similar pattern of strong bradycardia and AV block was observed in mice expressing a dominant negative HCN4 subunit lacking channel conductance and completely lacking *I*_*f*_. However, no mortality was observed in these mice, despite the presence of recurrent ventricular arrhythmia (Mesirca et al., 2014). Although HCN4 expression is essential for proper SAN functioning, extreme activation of *I*_*f*_ in cardiac cells can lead to initiation of ectopic foci, resulting in atrial and ventricular arrhythmias (Stieber et al., 2004). Morris et al. isolated atrial pacemaker cells from rats and found that overexpression of Hcn2 via adenovirus-mediated gene transfer resulted in pacing acceleration. This potentially implicates gain-of-function variants in *HCN2* and *HCN4* with increased pacemaker activity resulting in ectopic foci and cardiac arrhythmia (Morris et al., 2013). Interestingly, an Hcn1-deficient mouse model also showed bradycardia, slowed SAN conduction, sinus arrhythmia, and sinus pauses. This study suggests a role of *HCN1* in SAN function and human SND (Fenske et al., 2013). Although the phenotypes for *HCN4* variants in humans are varied, they nicely represent the various phenotypes in Hcn4-deficient mouse models.

### Sodium/Calcium Exchanger 1 Precursor (*SLC8A1*)

#### Background

Spontaneous action potentials in SAN cells provide the primary pacemaking activity for the entire heart and are important for proper cardiac functioning. The cardiac Na^+^-Ca^2+^ exchanger (NCX1) plays an integral role in diastolic depolarizations that trigger these recurrent action potentials. Following diastolic SR Ca^2+^ release from ryanodine receptors, increased cytosolic Ca^2+^ causes an inward current via NCX, which accelerates late diastolic depolarization to the action potential threshold (“calcium clock” model for pacemaker automaticity) (Bogdanov et al., 2001; Lakatta et al., 2010). Further, NCX1 inactivation has the ability to completely halt SAN firing by generating intermittent burst firing induced by intracellular Ca^2+^ overload (Groenke et al., 2013; Torrente et al., 2015). NCX1 has 10 transmembrane helices and four ion-binding sites, one for Ca^2+^ and three for Na^+^ (Liao et al., 2012; Secondo et al., 2015). This NCX1 structural study agrees with current stoichiometric studies showing an exchange rate of mostly three Na^+^ to one Ca^2+^ (Bers and Ginsburg, 2007). NCX1 can function to facilitate either inward or outward current depending on the membrane potential (Bers, 2002). NCX1 is the predominant pathway for calcium extrusion in cardiomyocytes during resting membrane potential; the high extracellular sodium concentration allows NCX1 to exchange calcium out of the cell. Overall, NCX promotes myocytes to relax, therefore implicating a role of NCX1 in contractility. Further, spontaneous pacemaker release of Ca^2+^ by RyR2 activates the NCX1 on the SR membrane, which then pushes the cell to the minimum threshold for triggering an action potential (Shattock et al., 2015).

#### Human Studies

Genetic variants in *SLC8A1*, the gene encoding NCX1, are associated with numerous electrocardiographic traits due to changes in calcium cycling. Polymorphisms in *SLC8A1* across human populations were first associated with hypertension. Seven NCX1 polymorphisms with a high minor allele frequency of more than 4% were identified in 1,865 individuals, with 787 being hypertensive (Kokubo et al., 2004). Hong et al. (2014) found a single-nucleotide polymorphism on the *SLC8A1* locus correlated with PR interval prolongation by a genome-wide association study using the Korea Association Resource database. Kim et al. (2012) also found a common *SLC8A1* variant associated with prolongation in QT interval, suggesting the predisposition of these populations to ventricular arrhythmias and SCD. Further, patients homozygous for allele rs13017968 in *SLC8A1* had higher rates of coronary artery abnormalities, predisposing these populations to Kawasaki disease. At present, there are no human data correlating mutations on *SLC8A1* with SND. However, animal models showed major phenotypes of SND when experiencing loss of NCX1 (Gao et al., 2013; Groenke et al., 2013; Torrente et al., 2017).

#### Mouse Models

The role of NCX1 in calcium cycling and its importance to the SAN have been studied through multiple *Slc8a1*-null mouse models. A global *Slc8a1* knockout resulted in abnormal myofibrillar organization and severe electrical defects that caused embryonic lethality (Fu et al., 2010); therefore, cardiac-specific knockouts are required to study the role of NCX specifically in the heart. Gao et al. (2013) used a global myocardial and SAN-targeted knockout of *Slc8a1* to study the role of NCX1 in pacemaker activity. Although isolated SAN cells showed similar basal contractility rates in *SLC8A1* knockout versus WT mice, *Slc8a1* knockout mice showed the inability to respond to isoproterenol, implicating a role of NCX1 in the sympathetic response of the heart (Gao et al., 2013). Several studies have established the role of NCX1 in calcium efflux; however, the role of NCX1 in triggering an action potential is still not completely understood. Groenke et al. (2013) showed pacemaker activity to be completely ablated in an atrial-specific *Slc8a1* knockout mouse model. The *Slc8a1* atrial specific knockout mouse also lacked P waves and had arrhythmic depolarizations in the SAN. Although SAN automaticity still occurred without NCX1, the automaticity came in bursts similar to tachycardia–bradycardia syndrome and SND (Torrente et al., 2015). Torrente et al. (2015) further used their atrial-specific *Slc8a1* knockout and found severe cellular Ca^2+^ accumulation during SA nodal pacemaker activity, leading to intermittent hyperactivation of small conductance K^+^ (SK) channels, subsequently resulting in arrhythmias. These data identified the potential influence of intracellular Ca^2+^ on SK channels and overall SAN repolarization and signified SK channels as potential therapeutic targets for SAN dysfunction if presented alongside Ca^2+^ cycling issues (Torrente et al., 2017).

### Ankyrin-2 (*ANK2*)

#### Background

Ankyrin-B (AnkB, encoded by *ANK2*) is a membrane adapter protein critical in the recruitment, organization, and stabilization of the ion channels and transporters underlying the excitation–contraction coupling, particularly in the SAN. Loss-of-function variants in *ANK2* are associated with a complex cardiac phenotype including heart rate variability, CPVT, conduction defects, AF, sinus node bradycardia, SCD, and, recently, arrhythmogenic cardiomyopathy (Roberts et al., 2019).

#### Human Studies

Two families characterized with severe SND were found to have *ANK2* allele variants, making AnkB the first non-ion channel protein associated with human SND (Curran and Mohler, 2011). Interestingly, individuals with AF have reduced levels of AnkB expression and increased levels of miR-34a (a microRNA associated with cardiac fibrosis). Of note, the 3′ untranslated region of *ANK2* also contains the binding site to miR-34a, implicating a potential role of miR-34a in the electrical remodeling of the atria and in the regulation of AnkB expression (Zhu et al., 2018). Although the culmination of cardiac AnkB studies implicate loss-of-function *ANK2* variants in numerous cardiac diseases, the lack of family history in many of these cases, and overall incomplete penetrance of AnkB-associated disease, strongly implies that additional genetic and/or environmental factors must be involved in the development of the severe “AnkB syndrome” phenotype. Notably, intense endurance exercise or other genetic variants likely play a role in the development of cardiac disease associated with loss-of-function *ANK2* variants (Roberts et al., 2019).

#### Mouse Models

Optical mapping was further used to analyze the complete, intact, atrial pacemaker complex. *Ank2*^+/−^ mice had greater RR variability due to multiple competing pacemaker sites between the SAN and the AV junction, further highlighting the role of AnkB in cardiac automaticity, yet suggesting some unknown mechanisms of compensation (Glukhov et al., 2010). SAN cells from *Ank2*^+/–^ mice showed reduction in membrane expression of NCX1, Na^+^-K^+^-ATPase (NKA), and voltage-dependent L-type calcium channel alpha1D subunit (Ca_v_1.3), causing abnormal intracellular Na^+^ and Ca^2+^ cycling, which generated various cardiac arrhythmic events (Curran and Mohler, 2011). Computational models have further analyzed the role of AnkB in the generation of lethal arrhythmias. The loss-of-function NCX and NKA specifically allows Ca^2+^ overload in the SR, therefore initiating afterdepolarizations and introducing variability and inconsistency in the SAN firing. Loss of Ca_v_1.3 in the SAN slows the overall pace of firing, explaining the bradycardia seen in families with AnkB dysfunction (Wolf et al., 2010, 2013).

### Myosin Heavy Chain 6 (*MYH6*)

*MYH6* encodes the alpha myosin heavy chain subunit of myosin (MHC-α), a major component of the sarcomere—a necessary component of muscle fiber for proper contraction in the heart (Epp et al., 1993; Squire, 1997). An MHC-α R721W missense variant has been identified in Icelandic populations (0.38% allelic frequency) and is associated with SND, with 50% of the carriers for this variant being diagnosed with SND. Carriers of the variant that were not diagnosed with SND still showed reduced heart rate and PR interval prolongation (Holm et al., 2011). Interestingly, another R654W heterozygous MYH-α variant was identified in an Australian family with severe yet diverse cardiac arrhythmias, including SND and cardiac arrest due to ventricular fibrillation, resulting in SCD or SND (Lam et al., 2015).

### Lamin A (*LMNA*)

Nuclear lamins (lamins A, B1, and B2) are the major components of nuclear lamina, which plays a vital structural role in the nuclear envelope (Mounkes et al., 2001). *LMNA* variants are associated with numerous cardiac conditions, particularly dilated cardiomyopathy (MacLeod et al., 2003; Lin et al., 2018; Yokokawa et al., 2019). A c.357-2A > G heterozygous splice site variant in *LMNA* was identified in a proband diagnosed with SND who had a family history of cardiac arrhythmia and dysfunction. This novel variant was predicted to cause haploinsufficiency, as aberrant mRNA from the mutant allele would likely decay by nonsense-mediated mRNA decay (Zaragoza et al., 2016). Although no population-wide variant in *LMNA* has been identified in relation to SND, the numerous familial variants identified and connected with conduction disorders provide a rationale for further exploration of the role of lamin A in SND.

### L-Type Calcium Channel Subunit Ca_v_1.3 (*CACNA1D*)

#### Background

Ca_v_1.2 (alpha1C) and Ca_v_1.3 (alpha1D) subunits make up the cardiac L-type voltage-activated calcium channel (Matthes et al., 2004). Ca_v_1.3 is expressed mostly in the SAN, AV node, and atrial myocytes (Mangoni et al., 2003). In the SAN, Ca_v_1.3 plays a major role in pacemaker activity by driving inward current during diastolic depolarization and regulating diastolic SR Ca^2+^ release (Mangoni et al., 2003; Torrente et al., 2016).

T-type calcium channels are composed of three subunits, Ca_v_3.1, Ca_v_3.2, and Ca_v_3.3, which are encoded by three genes: *CACNA1G*, *CACNA1H*, and *CACNA1I*. The three Ca_v_ subunits are responsible for the generation of T-type/low-voltage activated calcium currents (T-current) (Huc et al., 2009). Variants in *CACNA1H* and *CACNA1G* were identified in epileptic patients (Chen et al., 2003; Singh et al., 2007). Interestingly, earlier studies reported the expression of T-type calcium current (*I*_*Ca,T*_) in pacemaker cells and proposed its contribution to pacemaking function in SAN cells (Hagiwara et al., 1988). Later, Mangoni et al. (2006) demonstrated the direct contribution of Ca_v_3.1 channels to pacemaking and cardiac conduction through genetic ablation of Ca_v_3.1 channels in mice. The genetic inactivation of Ca_v_3.1 channels caused a slowing of pacemaking function through a reduction of the slope of the diastolic depolarization in SAN cells (Mangoni et al., 2006).

#### Mouse Models

*cacna1d*^–/–^ mice have severe SND and AV second-degree block (Platzer et al., 2000; Zhang et al., 2002; Mesirca et al., 2016). In addition, Ca_v_1.3 also plays an important role in calcium homeostasis in the ear, as *cacna1d*^–/–^ mice experience deafness along with SND (Chu et al., 2007). The phenotype of SND-associated deafness was later identified in two related families with a Ca_v_1.3 variant (G403_V404insG). This rare combination of symptoms is termed sinoatrial node dysfunction and deafness (SANDD) (Baig et al., 2011). This *CACNA1D* variant was also identified in four additional families with SANDD, along with an A376V missense variant (Liaqat et al., 2019).

### Short-Stature Homeobox 2 (*SHOX2*)

#### Background

Short-stature homeobox 2 is a transcription factor encoded by *SHOX2*. SHOX2 is essential for the proper development of the SAN (Puskaric et al., 2010). *Shox2*^–/–^ mice are embryonically lethal due to lack of SAN development, but *Shox2*^–/–^ zebrafish survive with bradycardia (Hoffmann et al., 2013). Mutations in genes encoding subunits and/or proteins directly involved or being components of the cardiac conduction system were linked to rhythm abnormalities (Frank et al., 2012). Interestingly, mutations in TBX genes such as *TBX3* and *TBX5* were associated with conduction dysfunction (Wolf and Berul, 2006; Christoffels et al., 2010). Notably, the transcription factor *NKX2-5* has also been identified as a regulator of heart rate, and several variants linked to *NKX2-5* were shown to cause heart rate variability and increased incidence of AF (den Hoed et al., 2013; Huang et al., 2013). Finally, transcriptome analysis of the SAN cells demonstrated a conserved genetic program between mouse and human cells including TBX3, SHOX2, ISL1, BMP, and NOTCH signaling components (van Eif et al., 2019).

#### Human Studies

Loss-of-function variants in *SHOX2* cause early-onset and familial AF (Hoffmann et al., 2016; Li et al., 2018). A heterozygous missense P33R SHOX2 variant was also identified recently in a patient with SND (Hoffmann et al., 2019). Screening should continue for *SHOX2* variants in patients with SND, as this gene has only been recently implicated in SAN function in humans.

### Transient Receptor Potential Cation Channel Subfamily C Member 3 (*TRPC3*)

#### Background

TRPC channels (non-selective Ca^2+^-permeable cation channels) are thought to play an important role in store-operated Ca^2+^ entry (SOCE), described as Ca^2+^ influx to the sarcolemma after Ca^2+^ depletion, although the pathway is currently poorly understood (Wu et al., 2004; Ju et al., 2007). TRPC channels are activated when diacylglycerol is released from the plasma membrane via agonist binding to G protein-coupled receptors (Wu et al., 2004). All TRPC subtypes (1–7) are expressed in the SAN except subtype 5, but TRPC3 is the only subtype expressed on the membrane surface of the pacemaker cells (Ju et al., 2007).

#### Human Studies

Human studies about TRPC channels are greatly needed. Current TRPC3 research is almost completely focused on animal models. One study identified marked increases of leukocyte TRP channel mRNA in 47 patients with non-valvular AF, implicating upregulation of TRPC channels in AF (Düzen et al., 2017).

#### Mouse Models

Ca^2+^ entry through TRPC3 in the SAN seems to play a role in AF and sinus arrhythmia. *Trpc3*^–/–^ mice treated with angiotensin II had reduced incidence of AF compared with WT control mice during AF pacing (Ju et al., 2015). Trpc3 specifically has been shown to increase local Ca^2+^ release (LCR) and NCX current (*I*_*NCX*_) in mouse embryonic stem cell-derived cardiomyocytes, which resulted in increased spontaneous action potentials (Qi et al., 2016). Both of these studies support the hypothesis that Ca^2+^ entry via TRPC3 is a pro-arrhythmic pathway, which potentially causes increased pacemaker activity. TRPC3 is upregulated in AF patients and AF animal models (Harada et al., 2012). Finally, TRPC-3 channel upregulation also has been shown to cause an increased accumulation of collagen consistent with atrial fibrosis in mice (Han et al., 2020). FK506-binding protein 52 (FKBP52 or KBP4) has been identified as an interaction partner of TRPC3 and may be an important player in TRPC3-related therapy going forward. Downregulation of FKBP52 induced a Trpc3-dependent hypertrophic response in neonatal rat cardiomyocytes (Bandleon et al., 2019). Upregulation of FKBP52 may be able to reduce activity of the pro-arrhythmic TRPC3 pathway, but more studies are needed to understand this interaction. TRPC also has potential as a target for cardiac fibrogenesis (Yue et al., 2013). TRPC3 has also been implicated in cardiac hypertrophy, and gene deletion of Trpc3 and Trpc6 in mice protected against pressure-induced cardiac remodeling (Seo et al., 2014).

### Stromal Interaction Molecule 1 (*STIM1*)

#### Background

SOCE is an important pathway for Ca^2+^ reentry after calcium depletion, particularly in cardiac pacemaker cells. STIM1 is an endoplasmic reticulum Ca^2+^ censor essential to the SOCE pathway, implicating STIM1 in the calcium clock of SAN cells. Following Ca^2+^ depletion in the SAN cells, STIM1 localizes to the cell periphery, along with calcium release-activated calcium channel protein 1 (Orai1) (Liu et al., 2015). STIM1 and Orai1 channels are selectively expressed in SAN cells, and STIM1 is essential in SAN functioning.

#### Mouse Models

*Stim1* cardiac knockout mice showed SR calcium store depletion in SAN cells, resulting in SAN dysfunction (Zhang et al., 2015). STIM1-deficient mice experienced slowed heart rate after stimulation, sinus arrest, and extreme autonomic response to cholinergic signaling. Further, they also showed reduction in L-type Ca^2+^ current and enhanced NCX activity, linking STIM1 to more regulatory pathways in Ca^2+^ cycling in SAN cells than previously anticipated (Zhang et al., 2015). Beyond the SAN, STIM1 has been found to play an essential role in interatrial conduction via its expression in sinus cardiomyocytes from the SAN to the coronary sinus. Deletion of *Stim1* from coronary sinus cardiomyocytes slowed conduction across the atria and increased susceptibility to atrial arrhythmias in *Stim1* cardiac-specific knockout mice (Zhang et al., 2020).

### Potassium Two Pore Domain Channel Subfamily K Member 2 (KCNK2)

#### Background

TREK-1, or K2p2.1 (encoded by *KCNK2* gene), is a K^+^ channel with four transmembrane segments and two pore domains that are activated via membrane stretch and arachidonic acid, among other mechanisms (Fink et al., 1996; Lesage and Lazdunski, 1998; Maingret et al., 1999). K^+^ channels in cardiac tissue open to cause hyperpolarization and close during depolarization, therefore playing a crucial role in selecting the duration of an action potential (Snyders, 1999). Beyond membrane stretch and arachidonic acid, TREK-1 is regulated by polyunsaturated fatty acids, temperature, receptor-coupled second messenger systems, volatile anesthetics, neuroprotectant agents, and selective serotonin reuptake inhibitors (Goonetilleke and Quayle, 2012).

#### Mouse Models

TREK-1 is expressed ubiquitously in porcine heart, with elevated expression in atrial tissue. Atrial burst pacing (a simulation for AF) has shown the ability to reduce Trek-1 expression by 70% after just 7 days in the atria (Schmidt et al., 2014). Cardiac-specific Trek-1 mice-deficient mice experienced bradycardia and sinus pauses following induced stress. Moreover, isolated SAN cells in Trek1-deficient mice showed decreased background K^+^ current that caused abnormal cell excitability, confirming the role of TREK-1 in the cardiac action potential. Our group has showed that βIV-spectrin/TREK-1 complex expression was decreased in a canine model with pacing-induced heart failure and SAN dysfunction (Unudurthi et al., 2016).

### Transient Receptor Potential Melastatin 4 (*TRPM4*)

TRPM4 is a selective monovalent cation channel that allows flow of Na^+^, K^+^, Cs^+^, and Li^+^ (Guinamard et al., 2010), which is activated by intracellular Ca^2+^ (Launay et al., 2002). A study in 160 unrelated probands identified multiple *TRPM4* variants associated with right-bundle branch block and isolated AV block, signifying the role of *TRPM4* in cardiac conduction. Surprisingly, none of the patients with SND harbored *TRPM4* variants (Stallmeyer et al., 2012). However, Trpm4 is expressed in mouse SAN cells and is potentially implicated in heart rate rhythm regulation (Demion et al., 2007). Further, TRPM4 inhibition showed the ability to reduce the action potential rate by modulation of Ca^2+^-activated non-selective cation current in isolated mammalian right atrial cells (Hof et al., 2013). Therefore, patients with SND should continue to be screened for *TRPM4* variants in the future considering the involvement of TRPM4 involvement in cardiac conduction.

## Clinical Management of Sinoatrial Node Dysfunction

### Pharmacologic Approach

Many genetic forms of SND are chronic, yet symptoms are mild. Acute SND, however, can appear secondary to another condition, procedure, or disease, as mentioned previously. Acute SND presented as bradycardia or bradycardia/tachycardia syndrome is dangerous and can be managed with a small selection of pharmacological agents. Therapeutic control of SND is ideal because of the reduced cost and avoidance of surgical intervention. Current pharmacological options for acute management of bradycardia associated with SND include atropine, isoproterenol, aminophylline, or theophylline according to the American College of Cardiology/American Heart Association/Heart Rhythm Society (ACC/AHA/HRS) updated guidelines in 2019 (Kusumoto et al., 2019).

Atropine may successfully reverse an acute SND condition to a normal sinus rhythm (Schweitzer and Mark, 1980). Atropine shortens the sinus cycle length and the sinus recovery time of the SAN, therefore potentially treating bradycardia (Dhingra et al., 1976). The SAN response to atropine is bimodal, however, with slowing of the heart rate in small doses (<0.4 mg) and acceleration of heart rate in higher doses (Das et al., 1975). Atropine is also a suitable therapeutic approach for myocardial infarction-induced bradyarrhythmia at <0.8 mg. Overall, atropine intravenous treatment of 0.5–2 mg total (only up to 1 mg at a time per 3–5 min) seems to be the best therapy for treatment of bradycardia attributable to acute SND (Kusumoto et al., 2019). Atropine, however, should not be used to treat bradycardia in patients who have undergone heart transplant, as atropine treatment resulted in sinus arrest or AV block in 20% of heart transplant patients from one study (Bernheim et al., 2004). Importantly, isoproterenol does not have any clinical trials as a pharmacological therapy for SND patients and therefore should be used with caution, particularly when there is a concern for coronary ischemia (Kusumoto et al., 2019). There are some positive case studies exploring isoproterenol as a therapeutic measure for bradycardia (Sodeck et al., 2007; Herman and Zhou, 2011), but the outcomes have reported incidence of supraventricular tachycardia (Cossú et al., 1997). In summary, atropine is generally the best pharmacological treatment for acute bradycardia.

Acute bradycardia following heart transplant is a common outcome. As mentioned earlier, atropine is a poor choice for bradycardia therapy after heart transplant due to the potential for complete AV block and sinus arrest (Bernheim et al., 2004). In this scenario, theophylline has displayed the ability to reverse bradycardia in the majority of post-transplant patients, averting the need for pacemaker implantation (Bertolet et al., 1996; Kertesz et al., 2003). After heart transplant, relative bradycardia is often considered as a heart rate of less than 80 bpm, as postoperative patients have higher hemodynamic demand. The lack of parasympathetic input in a donor heart often results in SND (Woo et al., 2008). Bertolet et al. (1996) showed the mean heart rate of 29 patients suffering from bradyarrhythmias post-heart transplant was improved from (62 ± 7) to (89 ± 10) bpm when treated with theophylline (300 mg intravenous, 474 ± 99 mg/day orally) after 24 h of treatment (Bertolet et al., 1996). Aminophylline has also some supportive data, but theophylline is more supported across the literature (Kusumoto et al., 2019). Theophylline shows some promise in treating chronic SND as well (Alboni et al., 1991; Saito et al., 1993), but theophylline has numerous situational restrictions that prevent it from becoming the gold standard for chronic SND therapy. Theophylline should specifically be avoided in cases of bradycardia/tachycardia syndrome or in patients experiencing any ventricular ectopy (Ling and Crouch, 1998).

Beyond therapeutic purposes, atropine and isoproterenol can be used to evaluate the severity of asymptomatic sinus bradycardia. Interestingly, the chronotropic response to atropine or isoproterenol can identify patients in need of preventative pacemaker implantations (Vavetsi et al., 2008).

Finally, a study of 192 patients with SND showed that cilostazol was able to prevent permanent pacemaker implantation. Only 20.4% of patients receiving cilostazol needed a pacemaker implant, while 55.8% of patients not receiving cilostazol required a pacemaker. By increasing heart rate, cilostazol shows promise to be the first long-term pharmacological therapeutic for SND as many of the pharmacological options discussed earlier are only effective short-term and have negative long-term side effects (Sonoura et al., 2019). With Sonoura et al. (2019) publishing this study in 2019, long-term studies showing longer-term survivability over 5 or 10 years in patients using cilostazol would further support the use of this drug to manage SND.

### Temporary Pacemaking

When pharmacological therapeutics fail, temporary pacemakers are an alternative approach and can be implemented via a multitude of methods for care of acute bradycardia. Transcutaneous cardiac pacing is an external pacing method (prevents the risk of infection and other surgical complications) and is commonly used in response to patients with life-threatening bradycardia (Dalsey et al., 1984). Transcutaneous pacing is often used for patients in cardiac arrest and can be successful if used within the early phases of cardiac arrest (Noe et al., 1986). Transcutaneous pacing does, however, show success in reverting bradycardia in patients not in cardiac arrest too (Clinton et al., 1985). Transvenous pacemaker therapy is another temporary pacemaker option. Transvenous pacemakers can be implanted in the internal and external jugular, subclavian, brachial, and femoral veins, although the right internal jugular vein is the most preferred location for best access to the right ventricle (Gammage, 2000). The early studies of transvenous pacemaker therapy showed very high rates of pacemaker malfunction (as high as 43%) (Lumia and Rios, 1973). Even today, approaches for temporary transvenous cardiac pacing vary widely, so outcomes are difficult to compare across studies (Diemberger et al., 2020). However, a study in 2018 across more than 360,000 patients found complications for modern-day temporary transvenous pacing to be only about 4%, but with 37.9% of patients still needing permanent pacemaker implantation (Metkus et al., 2019). Overall, the ACC/AHA/HRS guidelines recommend transcutaneous pacing over transvenous pacemaker therapy due to the complications of the transvenous method when a patient is hemodynamically unstable and critically ill due to bradycardia (Kusumoto et al., 2019). This suggestion may be changed in the future with large group studies showing the success of transvenous pacing.

### Permanent Pacemaker Implant

In general, a permanent pacemaker implant should be avoided if possible to avert the potential procedural complications related to the implant and long-term management of the implant. Unfortunately, permanent pacemaker implantation may be necessary to prevent severe and life-threatening arrhythmias and should be considered with severe symptomatic sinus bradycardia and bradycardia/tachycardia syndrome (Kusumoto et al., 2019). For example, permanent pacemaking after Fontan operation, a surgery for children with only a single functional ventricle, often results in SND and requires permanent atrial pacing in 13–16% of patients (Cohen et al., 1998; Takahashi et al., 2009). Further, patients presenting with AF and significant atrial fibrosis may require a permanent pacemaker implant (Akoum et al., 2012). Atrial flutter, associated with SND, can also be used to predict the need for pacemaker implantation. In 211 patients following ablation, an atrial flutter length of more than 273 ms predicted the need for a pacemaker implantation (Sairaku et al., 2012).

### Atrial Ganglionated Plexus Modification

Permanent pacemaker implantation is still the current therapeutic suggestion for long-term sinus bradycardia by the AHA (Tracy et al., 2013). However, younger SND patients will on average require a longer life from the permanent pacemaker and, therefore, require lead and generator replacements, which increase risk of infection and surgical complications (Qin et al., 2017). Notably, the sympathetic and parasympathetic nervous systems modulate sinus rhythm via the atrial ganglionated plexi (GP). Zhao et al. (2015) used endocardial radiofrequency ablation in the GP to prevent parasympathetic nervous system hyperactivity and successfully increased resting heart rate in young SND patients.

## Future Implications

SND is a disorder caused by the inability of the heart to perform its pacemaking function (Semelka et al., 2013). It is manifested clinically as sinus bradycardia, sinus pause, or alternating bradyarrhythmias and tachyarrhythmias. Importantly, patients report chronotropic incompetence in response to stress or exercise (Semelka et al., 2013). Permanent pacemaker implantation is only recommended for symptomatic patients. However, there is no existing therapy to reverse the primary genetic cause in patients diagnosed with primary or chronic SND. Calcium-activated potassium channels (SK4) have been implicated in the automaticity of cardiomyocytes, yet Haron-Khun et al. (2017) showed that TRAM-34 (a selective blocker of SK4 channels) successfully reduced DADs and calcium transients in human-induced pluripotent stem cell-derived cardiomyocytes (hiPSC-CMs) from patients with CPVT2 from a CASQ2-D307H variant. When mice with the CASQ2-D307H knock in variant were injected with TRAM-34, electrocardiographic recordings showed reduced arrhythmias (and reduced severity of arrhythmias) at rest and during exercise. This was also shown in *CASQ2* KO mice. TRAM-34 and clotrimazole (SK4 inhibitors) therefore hold great therapeutic promise for human populations with loss-of-function *CASQ2* variants (Haron-Khun et al., 2017).

Non-functional cardiac funny current (*I*_*f*_) causes unusual Ca^2+^ handling as previously discussed, therefore disrupting the pacemaker activity by the sinus node. Remarkably, Mesirca et al. (2014) showed how cardiac funny current-deficient mice have impulse generation and conduction defects, which can be rescued with genetic deletion of cardiac muscarinic G-protein-activated channels (GIRK4). Although *HCN4* and *GIRK4* loss-of-function variants have each been implicated in SND, the combination of silencing both genes seems to repair severe cardiac arrhythmia phenotype of SND associated with AV block and ventricular arrhythmia (Mesirca et al., 2014). Interestingly, follow-up work has indicated that genetic deletion of GIRK4 also rescues conduction defects in model mice of Ca_v_1.3-mediated SND (Mesirca et al., 2016). Furthermore, rescuing of SND in mice models carrying dysfunction in HCN4, Na_v_1.5, and Ca_v_1.3 channels can be mimicked by acute administration of the *I*_*KACh*_ blocker tertiapin-Q (Bidaud et al., 2020). Importantly, the ability of pharmacologic inhibition of *I*_*KACh*_ to improve SAN function has been demonstrated recently in human SANs with history of SND (Li et al., 2017). Finally, silencing of GIRK4 expression in human atrial myocytes was shown to efficiently decrease *I*_*KACh*_ densities and therefore is a great potential tool for treating arrhythmia (Liu et al., 2009). In conclusion, data from murine models and human SAN tissues indicate that gene therapy or pharmacologic strategy targeting GIRK4 channels can constitute an important future direction for clinical management of SND.

*HCN4* lentiviral gene transfer has interestingly shown the bioengineering potential to allow for pacemaker cell therapy. Transducing *HCN4* revived autonomous pacemaking and increased responsiveness to autonomic regulation in *HCN4*-transduced myocytes (Boink et al., 2008). Further, myocyte enhancer factor-2 (MEF2) and activator protein-1 (AP1), with binding sequences located on conserved non-coding sequence 13 (CNS13), are involved in HCN4 enhancement via the *HCN4* promoter (Kuratomi et al., 2009) and could be used to upregulate HCN4 to promote pacemaking activity.

In summary, SND is a disorder that more commonly affects the elderly population and impacts the pacemaking function of the heart resulting in arrhythmia and chronotropic incompetence. The complexity of SND is partially attributed to the complexity of genetic abnormalities and partially attributed to gene pleiotropy. Many variants of SND-associated genes can exhibit multiple unrelated phenotypic traits. Currently, clinical management of SND patients is restricted to the treatment or relief of arrhythmia symptoms. However, there is no widely available therapeutic option that targets or reverses the primary genetic cause in patients with chronic SND. Understanding the complexity of genetics that contribute to disease progression is critical to developing new therapeutic strategies for this complex, life-threatening disorder.

## Author Contributions

All authors listed have made a substantial, direct and intellectual contribution to the work, and approved it for publication.

## Conflict of Interest

The reviewer HZ declared a past co-authorship with the authors PM and MM to the handling editor. The remaining authors declare that the research was conducted in the absence of any commercial or financial relationships that could be construed as a potential conflict of interest.
